# Possibilities of Dementia Prevention - It is Never Too Early to Start

**DOI:** 10.25122/jml-2019-0088

**Published:** 2019

**Authors:** Sandra Morovic, Hrvoje Budincevic, Valbona Govori, Vida Demarin

**Affiliations:** 1. Aviva Medical Center, Zagreb, Croatia; 2. University Hospital Sveti Duh, Croatia, Zagreb; 3. Department of Neurology, University Clinical Center, Prishtina, Republic of Kosovo.; 4. International Institute for Brain Health, Zagreb, Croatia

**Keywords:** Alzheimer’s Dementia, Vascular Dementia, Cognitive Impairment, Dementia Risk Factors, Dementia Prevention

## Abstract

Dementia represents one of the greatest global challenges for health and social care in this century. More than 50 million people worldwide suffer from dementia, and this number is predicted to triple by 2050. Ageing is often associated with cognitive impairment. Therefore, prevention of cognitive impairment is an imperative. Dementia includes a heterogeneous group of disorders, the most common being Alzheimer’s disease and vascular dementia. Most cardiovascular risk factors such as hypertension, diabetes mellitus, hypercholesterolemia, atrial fibrillation and smoking are not exclusive risk factors for vascular dementia but also for Alzheimer’s disease. The ApoE4 allele is the single non-modifiable risk factor for Alzheimer’s disease. Today we know that an important, modifiable risk factor is education. Better education means better protection against dementia. A large number of dementia cases are potentially preventable by early intervention. Early changes in the blood vessel wall can be detected by early ultrasound methods or early biomarkers. These methods allow us to detect changes before the disease becomes clinically evident. Early disease detection enables timely management, and studies have shown that careful control of vascular risk factors can postpone the onset or even reverse disease progression.

## Introduction

### Possibilities of dementia prevention

Dementia represents one of the most significant global challenges for health and social care in this century. More than 50 million people worldwide have dementia, and this number is predicted to triple by 2050. Aging is often associated with cognitive impairment; therefore, prevention of cognitive impairment is imperative. Dementia includes a heterogeneous group of disorders, the most common being Alzheimer’s disease and vascular dementia. Most cardiovascular risk factors such as hypertension, diabetes mellitus, hypercholesterolemia, atrial fibrillation, and smoking are not exclusive risk factors for vascular dementia but also Alzheimer’s disease. The ApoE4 allele is the single non-modifiable risk factor for Alzheimer’s disease. Today we know that a significant, modifiable risk factor is education. Better education means better protection against dementia. A large number of dementia cases are potentially preventable by early intervention. Early changes in the blood vessel wall can be detected by early ultrasound methods or early biomarkers. These methods allow us to detect changes before the disease becomes clinically evident. Early disease detection enables timely management, and studies have shown that careful control of vascular risk factors can postpone the onset or even reverse disease progression.

The global aging population is leading to an increased number of older people, owing to factors such as increasing life expectancy and decreased birth rates. Aging is often associated with cognitive changes, which range from mild cognitive changes to severe dementia. This growing number of dementia suffering patients represents the most significant challenge for health and social care in this century. Although Alzheimer’s disease is the most common cause of a cognitive decline in the aged population, independent causes of cognitive dysfunction, such as vascular disease, subclinical brain injury, silent brain infarction, and clinically overt stroke, are important causes and contributors to the cognitive dysfunction [1,2].

Due to the unrecognized seriousness of the situation, The World Health Organization requested a draft global action plan on the public health response to dementia [3]. They defined dementia as an umbrella term for several diseases that are mostly progressive, affecting memory, other cognitive abilities, and behavior that interfere significantly with a person’s ability to maintain the activities of daily living. Dementia is a significant cause of disability and dependency among older people worldwide, having a significant impact not only on individuals but also on their careers, families, communities, and societies. Dementia accounts for 11.9% of the years lived with disability due to a non-communicable disease. In light of the improved life expectancy globally, this figure is expected to increase further. What is pointed out is the large gap between the need for prevention, treatment and care for dementia and the actual provision of these services. Dementia is under-diagnosed worldwide, and, if a diagnosis is made, it is typically at a relatively late stage in the disease process.

Long-term care pathways (from diagnosis until the end of life) for people with dementia are frequently fragmented, if not entirely lacking. The human rights of people with dementia are frequently denied in both the community and care homes. In addition, people with dementia are not always involved in decision-making processes, and their wishes and preferences of the provided care are often not respected. This is partly due to the late diagnosis, when individuals are no longer able to participate in the decision-making process due to the progression of the disease. The vision is clear: to have a global action plan for a world in which dementia is preventable, and people with dementia and their carers live well. Dementia patients receive the care and support they need to fulfill their potential with dignity, respect, autonomy, and equity. The goal is to improve the lives of people with dementia, their careers, and families while decreasing the impact of dementia on them as well as on communities and countries [3]. We hope to achieve these goals by 2025.

### Dementia and vascular risk factors

Dementia is a clinical syndrome characterized by the impairment of cognitive functions, such as memory, language, praxis, recognition, and executive function, with the loss of functional capacity [4]. Dementia may be caused by a heterogeneous group of disorders, the most common being Alzheimer’s disease (AD) and vascular dementia (VaD). While cardiovascular risk factors, such as diabetes mellitus, hypertension, hypercholesterolemia, atrial fibrillation, and smoking, are particularly relevant in the development of VaD, they also play a role in AD [5-10]. Thus, both conditions may represent different spectrums of cerebral vascular disease, depending on the extent of microvascular changes [11].

An association between impaired function of cerebral microvessels and cognitive impairment in patients with mild to moderate AD was shown in a study by Silvestrini [12]. Because some cardiovascular risk factors are modifiable, investigating the mechanisms by which they contribute to AD pathology and the manifestations of dementia may have implications for prevention. Also, lifestyle factors at a younger age, such as physical, cognitive and work activity, mild to moderate alcohol consumption, and diet may be protective against AD [13].

### Cognitive impairment

Mild cognitive impairment (MCI) is a clinical state of mild but abnormal memory loss without significant impairment in daily activities. In many instances, both clinically and pathologically, MCI represents a prodromal stage of Alzheimer’s disease. Some studies have shown that the presence of MCI in older subjects, regardless of the definitions and criteria used, increases the risk of developing dementia [14,15]. Because the cerebrovascular disease can cause mild cognitive deficits that affect multiple cognitive functions, the term ‘vascular’ mild cognitive impairment (VaMCI) was proposed [16,17]. Patients diagnosed with VaMCI are in transition toward Alzheimer’s disease [18]. Vascular cognitive impairment (VCI) encompasses all cognitive disorders associated with cerebrovascular disease, from developed mild cognitive deficits to dementia. VCI is a syndrome with evidence of clinical stroke or subclinical vascular brain injury and cognitive impairment affecting at least one cognitive domain. The most severe form of VCI is VaD [1,2].

### Less known risk factors

Apart from the recognizable vascular risk factors mentioned above, there might be one we are not considering. The ARIC (Atherosclerosis Risk In Communities) Study, with a 24-year follow-up (1987-2013) showed orthostatic hypotension to be a vascular risk factor for dementia. Orthostatic hypotension at midlife is an independent risk factor for incident dementia and ischemic stroke. The authors hypothesize the association between orthostatic hypotension and dementia is chronic cerebral hypoperfusion, which may have long-term effects on cognitive function. Similar explanations apply to studies linking orthostatic hypotension to obstructive sleep apnea or chronic obstructive pulmonary disease and increased risk for cognitive decline. Since half of the dementia cases are classified as Alzheimer’s disease, it is not surprising that the association of orthostatic hypotension and Alzheimer’s dementia is only half as strong as for the stroke. This again suggests that Alzheimer’s disease and vascular dementia share the same risk factors [19].

Divergent results were obtained by studies evaluating antihypertensive therapy in midlife. While treatment of hypertension is protective against stroke, further studies are needed to evaluate its effect on the development of dementia, since hypotension in later life is, on the other hand, also a risk factor for dementia [20].

### Clinical evaluation of dementia

The diagnostic evaluation of dementia is complex, with various criteria applied. The clinical diagnosis depends on the definition of the cognitive deficit and the differentiation between normal age-related changes and pathological conditions. Because the variability of cognitive function among the elderly is great, it is challenging to quantify precise normative limits in this population group [21].

The clinical diagnosis of dementia should always take into account the individual’s decline from the premorbid level of functioning [21]. In addition to the difficulty in differentiating between early AD and normal aging, the distinction among the various dementing illnesses presents a diagnostic challenge.

Recently, the Lancet Commission presented a new life-course model showing potentially modifiable and non-modifiable risks for dementia [2, 22]. According to this model, it is estimated that 35% of dementia cases could be prevented if we eliminate risk factors. A key recommendation is to focus on interventions to build up resilience and brain reserve, to activate neuroplasticity, detect and to treat risk factors and to live a healthier lifestyle. This life-course model shows us the need for preventive actions from early childhood, or even from birth.

As we already know, the most common form of dementia, Alzheimer’s disease, is, in large part, modulated by genetics. Genetics is one non-modifiable risk factor, meaning that with birth, we have either already inherited the ApoE4 allele, or not. Along with other genetic markers, the ApoE4 is among the few non-modifiable risk factors.

All other risk factors are potentially modifiable risk factors, and they account for about 35%. In early childhood, we need to start taking care of another risk factor for dementia, and that is education. Studies have shown that lower grade of education brings a higher risk for dementia, pointing to the conclusion that education protects against the onset of dementia. Education also influences the course and outcome of the disease in terms of a pattern of cognitive decline and underlying brain pathology. Study shows that the complexity of the adult life work and social network, with complex leisure activities reduce the occurrence of dementia complaints [2,22].

When we reach the midlife, at the age of 40 or 45 (45 is the official midlife beginning age), we are at risk of developing hearing loss [23], hypertension and obesity, the three health risks that if lasting for the rest of our middle age or longer, increase the risk for developing dementia [22]. So, keeping fit, taking care of the extra weight, as well as early recognition and treatment of hypertension will guard not only our body against diseases but also our brain.

The potential public health impact of hearing loss in the context of dementia is substantial, given the high worldwide prevalence of hearing loss in older adults. What we urgently need is an interdisciplinary effort to bring together hearing and mental health and to investigate further early hearing loss in the context of brain and cognitive aging [23]. In adult life, we need to take care of smoking, depression, physical inactivity, social isolation, and diabetes [22, 24-26].

Recently, Jaroudi and al. pointed toward the role of the hippocampus as the critical region affected by cognitive decline. The authors point out three major factors as personality, mood, and lifestyle. Personality traits and mood can be a positive or negative influence on memory, probably by having a positive or negative influence on the hippocampus function. The delay or the onset of neurodegenerative diseases (dementia) can be determined by the influence that these factors have on the course of the disease. Some recommendations exist regarding the living environment of the elderly, their nutrition, fitness, and emotional care. The importance of language is pointed out, not only in the sense of education and vocabulary but also in the complex properties of different languages [27].

The National Academy of Sciences reported in 2017 that there are no specific interventions that have sufficient evidence to warrant a public health campaign for the prevention of dementia except cognitive training, blood pressure management in people with hypertension, and increased physical activity [28,29]. In 2017, the presidential advisory from the American Heart Association/American Stroke Association tried to decide on a definition of initial optimal brain health in adults [21]. The working group identified seven metrics to define optimal brain health in adults, and these originated from well-known “Life’s simple 7” [30], identified by Ralph Sacco in 2011. He then identified four ideal health behaviors that are nonsmoking, physical activity, healthy diet, and a body mass index less than 25 kg/m^2^, and three ideal health factors such as untreated blood pressure less than 120/80 mmHg, untreated total cholesterol less than 200 mg/dL and fasting blood glucose less than 100 mg/dL. Along with these recommendations in order to maintain cognitive health, it is advised to incorporate control of cardiovascular risks and suggest social engagement and other related strategies. There is always an opportunity to improve brain health through adult prevention and other interventions.

Overall, white matter fiber-tracking on MRI evidenced an early signature of damage in hypertensive patients when otherwise undetectable by conventional neuroimaging. In perspective, this approach could allow identifying those patients that are in initial stages of brain damage and could benefit from therapies aimed at limiting the transition to dementia and neurodegeneration [31]. In adults with a high baseline blood-pressure, individuals using any blood-pressure-lowering drug, regardless of the type of medication, had a reduced risk for developing all-cause dementia and Alzheimer’s disease compared with those not using the antihypertensive drugs. It is also interesting that this meta-analysis looked not only at dementia but also at Alzheimer’s disease specifically, and found a benefit of lowering the blood-pressure. This suggests that the onset of Alzheimer’s disease may be slowed through the treatment of high blood pressure [32].

Several studies showed that increased arterial stiffness has a higher value in predicting the cognitive decline in healthy subjects, than the blood pressure itself [33]. It is superior to blood pressure in predicting cognitive decline in all domains and in explaining the hypertension-executive function association. Arterial stiffness, especially in hypertension, may be a target in the prevention of cognitive decline [33].

Neuroinflammation and cerebrovascular dysfunction occur very early, and are present already during the presymptomatic stage of Alzheimer’s disease, contributing to the progression of the disease. A recent study demonstrated that Alzheimer’s disease is accompanied by changes in cerebral spinal fluid levels of neuroinflammatory and cerebrovascular biomarkers during the pre-clinical phase. These changes reflect the inflammatory and vascular dysfunction in the brain. These pathologic processes contribute to the pathology, neurodegeneration, cortical atrophy, and increased risk of AD [34].

The influence of hormones on cognition is also being investigated. A study comparing women who have given multiple births and women who had incomplete pregnancies on late-life cognition and the risk of developing Alzheimer’s disease was conducted. The connection has been made primarily with high levels of estrogen during pregnancy and its abrupt withdrawal after childbirth. Even though the results pointed to five or more pregnancies as being a higher risk, this area needs further investigation before reaching a solid conclusion [35].

Traumatic brain injury (TBI) alone is a risk factor for cognitive changes, but the risk increases 3 to 4 times in individuals who have diabetes. A study which included patients with type I diabetes who suffered a TBI, showed that TBI is a trigger for neurotoxic events, from acute (axonal shearing, vasospasm ischemia) to secondary (inflammation, oxidative stress, apoptosis) and chronic (increased levels of amyloid precursor protein, hyperphosphorylation of the tau protein). TBI disturbs the brain glucose metabolism which results in an increased demand for glucose in the first two days. This causes a hyperglycemia in almost half of TBI patients, and this is a risk factor for oxidative stress. Several of these events can elevate the risk of dementia, including inflammation, oxidative stress, and the production of beta-amyloid [36, 37].

An increasing number of studies confirm the positive correlation between obesity and inflammation with cognitive impairment [38, 39]. There is sufficiently strong evidence, from a population-based perspective, to conclude that regular physical activity [40] and management of cardiovascular risk factors (diabetes, obesity, smoking, and hypertension) [41] reduce the risk of cognitive decline and may reduce the risk of dementia. Also, there is sufficiently strong evidence to conclude that a healthy diet and lifelong learning/cognitive training may also reduce the risk of cognitive decline, thus enhancing the innate mechanism of neuroplasticity [42, 43].

Findings indicate that older men with a history of depression are at increased risk of developing dementia, although depression in later life is more likely to be a marker of incipient dementia than a modifiable risk factor. Older people with depression may be better viewed as potential targets of indicated prevention strategies, similar to people with mild cognitive impairment [24].

The window of opportunity for the beneficial effects of physical activity seems to be broad and may extend to people who become active later in life. The first trial to demonstrate the benefit of physical activity on cognitive function in older adults with subjective and objective mild cognitive impairment was completed ten years ago. It made clear that physical activity improves cognition, possibly by improving cerebral vascular function and brain perfusion. Another explanation is spending time in a stimulating, enriched environment associated with physical activity. Activity-prone environments contribute to enhanced brain plasticity by stimulating synaptogenesis, neurogenesis, and building up protection against stress. A human study demonstrated that physical activity is associated with increased blood perfusion of attention modulating brain regions [44].

However, beyond already available general recommendations for health promotion, it is very challenging to draw specific practical recommendations from the current evidence regarding the type, frequency, intensity, and duration of physical activity that could protect against AD. It is likely that physical activities that have additional social and cognitive stimulation components may be most effective. The multi-domain approach to dementia prevention also seems more promising compared with the traditional, single-domain approach [26].

When looking for preventive therapies beyond the officially accepted pharmacological treatment, studies have proven that adherence to the Mediterranean diet [45,46] led to a reduced beta-amyloid accumulation. Results have shown that the intake of fruits is probably one of the essential elements of the Mediterranean diet. An increased intake of fruit was associated with less accumulation of beta-amyloid. Even though the mechanism is unclear, it is probably associated with the polyphenolic content of fruits.

Smaller studies showed an improvement in cognitive tasks with the intake of two supplements. A randomized, double-blind, placebo-controlled study with Ginko-Biloba standardized extract showed beneficial effects in slowing down the cognitive deterioration in patients diagnosed with vascular cognitive impairment. However, this was a small study, and further investigation is needed [47].

Another randomized, double-blind, placebo-controlled trial in patients receiving NADH (nicotinamide adenine dinucleotide) showed no significant decline in cognitive function after six months, as opposed to a placebo-controlled group. The group taking NADH showed better performance in verbal memory, verbal fluency, as well as overall dementia rating. NADH is a natural substance present in all living cells, making it safe. It is well-tolerated, and caregivers (in previous trials) report good tolerance and no adverse events [48]. Perhaps these therapies could be considered more commonly in clinical practice, as mono- or add-on substances.

Loneliness predicted higher dementia risk, whereas being married and having many close relationships with friends and family members, proved to lower the risk of dementia. Further epidemiological research is needed to understand the possible causal nature of these associations, including the likely underlying mechanisms [49].

The Finnish Geriatric Intervention Study to Prevent Cognitive Impairment and Disability (FINGER) is the first multi-domain lifestyle intervention that has shown that a combination of lifestyle interventions can prevent or slow down cognitive decline [50]. Numerous evidence from epidemiological studies indicates that different modifiable lifestyle factors are related to dementia and Alzheimer’s disease. The intervention areas were diet (Nordic diet), exercise, cognitive training (individualized) and vascular risk monitoring. The results showed a reduction in cognitive decline by 30%. There are now three multi-domain trials going on. The FINGER study was taken as a model, and it included 1109 participants in the analysis: 362 APOE 4 allele carriers (173 interventions and 189 controls) and 747 non-carriers (380 interventions and 367 controls). The difference between the intervention and control groups in annual neuropsychological test battery total score change was 0.037 (95% CI, 0.001 to 0.073) among carriers and 0.014 (95% CI, 0.011 to 0.039) among non-carriers. The intervention effect was not significantly different between carriers and non-carriers (0.023; 95% CI, 0.021 to 0.067). Healthy lifestyle changes may be beneficial for cognition in older at-risk individuals, even in the presence of APOE-related genetic susceptibility to dementia.

Assuming a causal relation and intervention at the correct age for prevention, relative reductions of 10% per decade in the prevalence of each of the seven risk factors could reduce the prevalence of AD in 2050 by 8 to 3% worldwide. After accounting for dependence among the risk factors, around a third of Alzheimer’s disease cases worldwide can be attributed to potentially modifiable risk factors.

The incidence of Alzheimer’s disease can be reduced through improved access to education, reduction of vascular risk factors (through the use of effective methods such as physical activity, nonsmoking, diagnosing and treating midlife hypertension, obesity, and diabetes) and depression [45, 51].

## Conclusion

A new clinical methodology was introduced to evaluate and treat AD patients. Because AD starts at least twenty years before its clinical onset, we must target individuals at the beginning of middle age, if not earlier. Among individuals aged 85 or over, with AD (at this age, around 30% of individuals develop dementia due to AD), brain pathology started between the ages 55 and 65. Similarly, among individuals aged 65, who develop AD, brain pathology started between ages 35 and 45. This represents a change in which we view and define AD, shifting it from the disease of older age to the disease of the middle age or younger age. This is the prodromal phase called the prodromal AD, and is the period without evident cognitive symptoms, but offers an excellent opportunity for early interventions.

The general recommendation is to apply risk-free interventions with empirical evidence of efficacy, and without excessive expectations on the results. Clinical precision medicine uses expanded medical history, along with past medical history and physical (neurological) examination, and is then interpreted in conjunction with anthropometrics, blood biomarkers, and cognitive results. Each data point is taken into consideration, and the patient is followed longitudinally to evaluate the effectiveness of the intervention [52].

## Conflict of Interest

The authors confirm that there are no conflicts of interest.
